# Allogenic Adipose-Derived Mesenchymal Stem Cell Infusion for the Management of Acute-Onset Pancreatitis in Dogs: A Pilot Study

**DOI:** 10.3390/ani14192905

**Published:** 2024-10-09

**Authors:** Harry Cridge, Valerie Johnson

**Affiliations:** Department of Small Animal Clinical Sciences, College of Veterinary Medicine, Michigan State University, East Lansing, MI 28824, USA

**Keywords:** MSC, pancreatitis, cPLI, Spec cPL, refractory disease

## Abstract

**Simple Summary:**

Pancreatitis is a common cause of gastrointestinal upset in dogs. Despite its common occurrence, treatment options are limited, and veterinarians are often restricted to providing supportive and symptomatic care. In this study, we document the use of a mesenchymal stem cell (MSC) infusion in a small number of dogs with acute pancreatitis and compare it to a population of dogs receiving a routine standard-of-care treatment. In this pilot study, we found no significant differences in the measured biochemical or clinical score systems, but clinical improvements were noted in two dogs with previously refractory disease. While MSCs appear safe, larger trials are needed to investigate the use of this treatment in dogs with acute pancreatitis. MSCs may be considered by veterinarians for refractory disease, while additional data are pending.

**Abstract:**

Mesenchymal stem cells (MSCs) have significant anti-inflammatory properties and are beneficial in rodent models of pancreatitis. The safety and efficacy of MSCs is unknown in dogs with acute pancreatitis (AP). Dogs with AP who were treated with MSCs (*n* = 4) were identified prospectively for this pilot study from an academic hospital. Serum Spec cPL and C-reactive protein (CRP) concentrations were measured on the day of MSC administration and 2 days later. The clinical severity, via the Modified Clinical Activity Index (MCAI), was also calculated. Two dogs received MSCs shortly after AP diagnosis, while the remaining dogs received MSCs due to clinically refractory disease. Changes in Spec cPL, CRP, and MCAI in the MSC-treated dogs were compared to a control population (*n* = 7) receiving the standard-of-care treatment for AP. No significant differences were noted between the populations for changes in Spec cPL (*p* = 0.79), CRP (*p* = 0.67), or MCAI (*p* = 0.91). However, subjective clinical improvements were noted within 24 h of MSC infusion in the two dogs with previously refractory disease. MSC infusions appear safe in the management of AP in dogs and may be considered in refractory disease. However, given the nature of this pilot study and its limitations, larger randomized controlled clinical trials are needed to truly evaluate the efficacy of MSC infusions in dogs with AP.

## 1. Introduction

Acute-onset pancreatitis (AP) is a common inflammatory disorder in dogs, with a high mortality rate [1,2,3,4]. Treatment has long been limited to supportive and symptomatic care, which does not address the root cause of pancreatic inflammation. While fuzapladib sodium has been introduced to the United States market and improves the clinical signs of AP in dogs, anecdotes suggest that additional adjunctive modalities may be needed in severe cases of pancreatitis in dogs [5].

Mesenchymal stem cells (MSCs) have significant immunomodulatory and anti-inflammatory properties [6]. The therapeutic potential of systemically administered MSCs have been investigated in a large number of inflammatory conditions in both humans and companion animals; however, this treatment has not been investigated in dogs with pancreatitis [7,8]. Rodent models suggest that MSCs may play a role in the management of pancreatitis by reducing mortality, decreasing inflammatory biomarker concentrations, and ameliorating pancreatic pathology [9,10]. MSCs also have distant effects, thereby reducing the complications of pancreatitis, which could alter the prognosis [11]. The combination of the significant efficacy of MSCs in rodent models of pancreatitis in addition to their clinical safety and efficacy in dogs with other inflammatory conditions makes MSCs a prime therapeutic target in this common yet challenging disease process. Prior studies have documented the safety of MSC infusions in naturally occurring disease in dogs [12].

Thus, the aim of our pilot study was to describe the use of MSCs in dogs with naturally occurring AP. A secondary aim was to evaluate the safety and efficacy of MSCs in the management of AP in dogs.

## 2. Materials and Methods

### 2.1. Prospective Case Identification

All cases of pancreatitis (diagnostic criteria described below) that were known to the study investigators were offered voluntary enrollment in this prospective study. The study investigators were alerted to potential cases by staff veterinarians at the teaching hospital. The first four dogs with pancreatitis and informed owner consent were enrolled in this pilot study. A clinical diagnosis of pancreatitis was based on the integration of data from a history, physical examination, abdominal ultrasound, and the results of a pancreatic lipase assay [13]. Dogs were required to have ≥2 of the following clinical signs: hyporexia, vomiting, diarrhea, lethargy, and abdominal discomfort. Additionally, dogs were required to have ≥2 sonographic features of AP, including pancreatic enlargement, hypoechoic pancreatic parenchyma, and a hyperechoic surrounding mesentery [14]. All dogs were also required to have an above-reference-interval canine pancreas-specific lipase (Spec cPL) concentration. Once a case was determined to have met the above criteria, the owners were contacted for voluntary enrollment in the study and provided informed consent. All dogs in the prospective arm of the study received MSCs in a non-randomized fashion. The study protocol was approved by the Michigan State University IACUC committee.

### 2.2. Historic Control Group

A historic control population of 8 dogs who were previously managed for AP using the standard-of-care therapy was utilized. Eight dogs were screened to allow for an intended 2:1 ratio of control dogs to MSC dogs. Clinical data were available for these dogs. Standard-of-care therapy was defined as the provision of fluid therapy, opioid analgesia, enteral nutrition, and anti-emetic medications. No dogs in the control group received MSCs or fuzapladib sodium. The diagnosis of pancreatitis was confirmed utilizing the same criteria as the MSC-treated group. Spec cPL and CRP concentrations had been calculated at the time of treatment and were available within the available records. MCAI was similarly recorded. The same diagnostic criteria for pancreatitis (as defined in Section 2.1) were applied to the historic control group.

### 2.3. Culture of Adipose-Derived MSCs

Serum and whole blood from MSC donors were analyzed for infectious diseases including serology to test for *Anaplasma phagocytophilum, Borrelia burgdorferi, Dirofilaria immitis,* and *Ehrlichia canis* (4DX, IDEXX laboratories Inc, Westbrook, MA, USA), and PCR was performed to detect the presence of *Hemoplasma spp*, *Ehrlichia spp*, *Bartonella spp*, and *Rickettsial spp* (Michigan State University Veterinary Diagnostic Laboratory, Lansing, MI, USA). All donors had normal CBC and biochemistry profiles (BUN, crea, Na, K, Cl, HCO_3_, tCa, P, Mg, Alb, Glob, Glu, tbili, ALP, GGT, ALT, AST, CK, chol) and were negative on all infectious disease testing parameters.

Adipose tissue-derived MSCs were generated from adipose tissue that was aseptically collected from young healthy dogs undergoing a simple gastrotomy or enterotomy for the removal of foreign material with informed owner consent. To prepare the MSC cultures, adipose tissues were minced under sterile conditions, incubated in a 1 mg/mL solution of collagenase (Sigma-Aldrich, St. Louis, MO, USA) at 37 °C with 5% CO_2_ for 30 min, and then triturated. The cell suspension was then centrifuged at 1050× *g* to pellet the stromal vascular fraction and then resuspended in an MSC culture medium. The MSC culture medium consisted of low-glucose DMEM (InVitrogen/Gibco, Carlsbad, CA, USA) supplemented with essential and non-essential amino acids (InVitrogen/Gibco, Carlsbad, CA, USA), glutamine (InVitrogen/Gibco, Carlsbad, CA), 15% fetal bovine serum (Peak Serum, Wellington, CO, USA), and penicillin and streptomycin solution (InVitrogen/Gibco, Carlsbad, CA, USA). The cells in the medium were allowed to adhere in tissue culture flasks (BD Falcon, Bedford, MA, USA) for 72 h, after which the non-adherent cells were removed, and a fresh medium added. When the cells reached 80–90% confluence, they were passaged using a trypsin-EDTA solution (InVitrogen/Gibco, Carlsbad, CA, USA). Cells were then harvested following expansion between passages 3 and 5. The cells were placed in freezing media (92% fetal bovine serum, 8% DMSO—Sigma Aldrich, St. Louis, MO, USA) and slow-frozen over 24 h and subsequently stored in the vapor phase of liquid nitrogen. Prior to administration to dogs, these cells were thawed and put back into a culture for 24 h. This protocol has previously been utilized and reported by the authors [12].

### 2.4. Administration of MSCs and Safety Assessment

Dogs were administered MSCs intravenously at a dose of 2 × 10^6^ cells/kg in phosphate-buffered saline (PBS) over 15 min. Cells were resuspended in 10 mL of PBS for animals weighing 5–20 kg, 15 mL of PBS for animals weighing 20–30 kg, and 20 mL of PBS for animals >20 kg in weight. Heparin was added at 100 IU/10 mL just prior to injection based on the injection volume. This protocol has previously been utilized and reported by the authors [12].

The safety of the MSC infusion was evaluated via clinical assessment by a licensed veterinarian. The clinical assessment included repeat measurement of vital parameters (temperature, heart rate, and respiratory rate) every 15 min and monitoring for the development of urticaria or other signs of a hypersensitivity reaction.

### 2.5. Clinical Severity Index

The modified clinical activity index (MCAI) was utilized to assess the clinical severity of AP as previously described [15,16]. The MCAI was prospectively calculated on day 0 (enrollment) and 2 days later.

### 2.6. Laboratory Testing

Serum pancreatic lipase concentrations were quantified using a commercial assay (Spec cPL, Texas A&M University Gastrointestinal Laboratory, College Station, TX, USA). The assay has been validated for use in dogs and is unaffected by extra-pancreatic lipases [17,18]. Samples were collected prior to MSC infusion and 2 days later. Serum samples were immediately frozen and stored at −80 °F. The samples were submitted to the laboratory for analysis within 30 days of collection.

Serum CRP concentrations were quantified using a commercial assay (Gentian canine-specific immunoturbidometric CRP assay, Cornell University Animal Health Diagnostic Center, Ithaca, NY, USA). Samples were collected prior to MSC infusion and 2 days later. This assay is used as a biomarker of systemic inflammation and has previously been validated for use in dogs [19]. Serum samples were immediately frozen and stored at −80 °F. The samples were submitted to the laboratory for analysis within 30 days of collection.

These tests were performed prospectively in both groups.

### 2.7. Statistical Analysis

Continuous data sets were assessed for normality using Shapiro–Wilk testing. Data were reported as medians and interquartile range (IQR). Decreases in the Spec cPL, CRP, and MCAI concentrations from day 0 (day of MSC infusion) to day 2 were compared between the MSC-treated group and the control group using a Mann–Whitney U test. Statistical analyses were performed using commercially available software (GraphPad Prism Version 10.0, GraphPad Software Inc., San Diego, CA, USA). The significance was set at *p* < 0.05 for all statistical comparisons. Descriptive analyses were also performed.

## 3. Results

### 3.1. Animals—MSC Group

Four dogs were treated with an MSC infusion as part of this pilot study. The median age of the dogs in the MSC-treated group was 7 years (IQR: 9.6 years). The median weight was 12.2 kg (IQR: 7.0 kg). All dogs in the MSC-treated group were male and neutered. Two dogs were mixed breed, while one each of the following breeds were represented: Australian Shepherd and Pomeranian.

All four dogs received intravenous fluid therapy, opioid analgesia (fentanyl 3–5 μg/kg/m IV), anti-emetics (maropitant citrate at 1 mg/kg IV or 2 mg/kg PO q 24 h and/or ondansetron 0.2–0.5 mg/kg PO q 12 h), and enteral nutrition (voluntary intake, or ¼ resting energy requirement (RER) on day 1–2 of hospitalization, slowly increasing by ~25% RER every 24 h if well tolerated). Two dogs received the MSC infusion within 24 h of diagnosis of AP, while the remaining two dogs received MSCs after failing to respond to the standard-of-care treatment.

Of the two dogs that received MSCs shortly after AP diagnosis, one dog received MSCs alongside the standard-of-care therapy for AP, and the dog was clinically improved by the next day, as demonstrated by absent abdominal pain and the return of appetite. The second dog had been managed supportively for gastrointestinal upset (for 48 h) without improvement and had an abdominal ultrasound performed, confirming a diagnosis of pancreatitis, and received an MSC infusion due to continued anorexia despite supportive care. The dog was noted to be eating within 24 h of MSC infusion.

Of the two dogs that received an MSC infusion due to refractory pancreatitis, one dog had severe abdominal discomfort (despite methadone administration) and intractable regurgitation, which markedly improved within 24 h of MSC administration. Methadone was continued at the same dose. The second dog had failed to respond to standard-of-care therapy and three doses of fuzapladib sodium (8–11 days prior to MSCs). Its quality of life and potential euthanasia were considered (day 11 of hospitalization). This dog was noted to be subjectively brighter, subjectively more alert, and have an improved appetite within 24 h of MSC administration, as determined by the attending veterinarian. Caloric intake was not recorded. The dog was discharged from the hospital 3 days later. All four dogs survived to hospital discharge.

No acute hypersensitivity reactions were noted following MSC administration. One dog was noted to have a seizure during hospitalization; however, the dog had a prior history of seizures. One dog was noted to have a persistent long-standing mixed hepatopathy of unknown etiology. The biochemical evidence of a hepatopathy pre-dated MSC administration.

### 3.2. Animals—Control Group

Of the eight dogs in the control population, seven dogs met this study’s diagnostic criteria for pancreatitis. One dog was excluded due to a within-reference-interval Spec cPL concentration. The median age of the control group was 11 years (IQR: 12.5 years). The median weight was 6.3 kg (IQR: 7.2 kg). Four dogs were male and neutered, and three dogs were female and spayed. Three dogs were mixed breed, while one each of the following breeds were represented: Australian cattle dog, Bichon frise, Miniature Pinscher, and Yorskhire terrier. All seven dogs survived to hospital discharge.

### 3.3. Changes in Spec cPL, CRP, and MCAI

There was no significant difference in the median decrease in Spec cPL (*p* = 0.79) or CRP (*p* = 0.67) concentrations from day 0 (enrollment) to day 2 between the MSC-treated group and the control group. Similarly, there was no difference in the median decrease in MCAI between the MSC-treated group and the control group (*p* = 0.91) from day 0 (enrollment) to day 2.

## 4. Discussion

In this study, we document the use of allogenic adipose tissue-derived MSC infusions in the management of AP in dogs. Two of the treated dogs received the MSCs shortly following diagnosis, as an adjunctive agent to the standard-of-care treatment, while two of the treated dogs received the MSC infusion as a ‘rescue’ agent due to clinical severity and/or failure to respond to the standard-of-care treatment.

No acute adverse effects were noted following administration of the MSC infusion, and all dogs survived to discharge. Two dogs were thought to have continuations of pre-existent concurrent disease (seizures (*n* = 1) and mixed hepatopathy (*n* = 1)); however, while highly unlikely, it is not definitively possible to rule out MSCs as a contributing factor to these abnormalities. Prior studies have also documented the safety of the MSC protocol used in this study, including by one of the study investigators, in a study of elbow osteoarthritis in dogs [12].

In our study, the median change in Spec cPL and CRP from the day of MSC administration to two days later was not statistically different from the change in Spec cPL and CRP from day of pancreatitis diagnosis to two days later in the control population. However, this was likely influenced by the small population size and the use of MSC infusions in dogs that had poor responses to pre-existing treatment in two of the four dogs. Spec cPL was used as a biomarker of pancreatic inflammation, whereas CRP was utilized as a biomarker of systemic inflammation in our study. Similar results, albeit on a slightly different timeline (day 0 to day 3), were seen with fuzapladib sodium in a recent study [5]. In contrast to fuzapladib sodium, we did not note a difference in MCAI improvement in our study between the MSC (prospective) and control (retrospective) group, although this may have been influenced by the population size, selection bias, and different clinical timelines between the two studies [5]. Despite a lack of statistical improvement in MCAI between the two populations, it is of interest that two dogs with clinically severe/refractory disease had marked subjective improvements in clinical signs within 24 h of MSC infusion. These findings prompt further investigation of this treatment in larger-scale randomized controlled studies.

This study had limitations. As with all pancreatitis studies investigating treatment, dogs received a combination of medications, meaning that it is not possible to definitively assign differences in outcome between the two groups to the use of an MSC infusion. Additionally, this study is limited by a small population size within the MSC-treated group, which may have contributed to the lack of a significant difference in changes in Spec cPL, CRP, and MCAI between the populations. A sample size calculation was not utilized due to the limited data in this field. This pilot study will allow for power calculations for future larger studies. Additionally, MSCs are known to be a heterogenous cell population, and donor variability can contribute to variability in response to stem cell treatment. This variability was minimized by utilizing young healthy donors and using cells at a low passage. This study remains useful, however, in documenting the safety of MSCs in this population and documenting the subjective clinical improvement in two dogs with clinically refractory disease. This study/data may also act as a basis for hypothesis generation and power calculations for larger randomized controlled clinical trials involving MSC infusions in dogs with pancreatitis.

## 5. Conclusions

Allogenic adipose tissue-derived MSCs, administered as per the protocol in this study, appear safe for use in dogs with AP and could be considered in refractory cases of the disease; however, no statistically significant changes in measured data were noted. Randomized controlled clinical trials, with larger case numbers, are needed to truly investigate the efficacy of MSC treatment and to understand when it is best to use this treatment in dogs with AP.

## Data Availability

Data are available from the authors upon reasonable request. Data are not available publicly to maintain case anonymity given the small population size.
